# A Hypothesis for the Evolution of Nuclear-Encoded, Plastid-Targeted Glyceraldehyde-3-Phosphate Dehydrogenase Genes in “Chromalveolate” Members

**DOI:** 10.1371/journal.pone.0004737

**Published:** 2009-03-09

**Authors:** Kiyotaka Takishita, Haruyo Yamaguchi, Tadashi Maruyama, Yuji Inagaki

**Affiliations:** 1 Japan Agency for Marine-Earth Science and Technology (JAMSTEC), Yokosuka, Kanagawa, Japan; 2 Graduate School of Life and Environmental Sciences, University of Tsukuba, Tsukuba, Ibaraki, Japan; 3 Center for Computational Sciences and Institute of Biological Sciences, University of Tsukuba, Tsukuba, Ibaraki, Japan; East Carolina University, United States of America

## Abstract

Eukaryotes bearing red alga-derived plastids — photosynthetic alveolates (dinoflagellates plus the apicomplexan *Toxoplasma gondii* plus the chromerid *Chromera velia*), photosynthetic stramenopiles, haptophytes, and cryptophytes — possess unique plastid-targeted glyceraldehyde-3-phosphate dehydrogenases (henceforth designated as “GapC1”). Pioneering phylogenetic studies have indicated a single origin of the GapC1 enzymes in eukaryotic evolution, but there are two potential idiosyncrasies in the GapC1 phylogeny: Firstly, the GapC1 tree topology is apparently inconsistent with the organismal relationship among the “GapC1-containing” groups. Secondly, four stramenopile GapC1 homologues are consistently paraphyletic in previously published studies, although these organisms have been widely accepted as monophyletic. For a closer examination of the above issues, in this study GapC1 gene sampling was improved by determining/identifying nine stramenopile and two cryptophyte genes. Phylogenetic analyses of our GapC1 dataset, which is particularly rich in the stramenopile homologues, prompt us to propose a new scenario that assumes multiple, lateral GapC1 gene transfer events to explain the incongruity between the GapC1 phylogeny and the organismal relationships amongst the “GapC1-containing” groups. Under our new scenario, GapC1 genes uniquely found in photosynthetic alveolates, photosynthetic stramenopiles, haptophytes, and cryptopyhytes are not necessarily a character vertically inherited from a common ancestor.

## Introduction

Glyceraldehyde-3-phosphate dehydrogenase (GAPDH) is an ubiquitous enzyme catalyzing the reversible interconversion between glyceraldehyde-3-phosphate and 1,3-diphosphoglycerate. GAPDH gene sequences are available for diverged eukaryotes, and intensive phylogenetic investigations have revealed a complex evolution of GAPDH genes in photosynthetic eukaryotes. Photosynthetic eukaryotes generally possess two different types of GAPDH genes in their nuclear genomes. One of the two GAPDH enzymes works in the cytosol and is involved in glycolysis/gluconeogenesis, while the other is targeted to plastids and catalyzes Calvin cycle reactions. In land plants, green algae, red algae, glaucophytes, and euglenids, plastid-targeted GAPDH enzymes bear a clear evolutionary affinity to cyanobacterial homologues (so-called GapA/B), and are distantly related to cytosolic enzymes (so-called GapC). These findings suggest that an ancestral GapA/B gene was acquired from an endosymbiotic cyanobacterium that gave rise to plastids, being phylogenetically distinctive from the cytosolic counterpart [1]–[5]. In sharp contrast, all known photosynthetic eukaryotes with red alga-derived plastids (*Chromera*, the vast majority of photosynthetic dinoflagellates, photosynthetic stramenopiles, cryptophytes, and haptophytes) as well as the apicomplexan parasite *Toxoplasma* utilize GapC-related enzymes for plastids instead of GapA/B [6]–[12]. Henceforth, we designate nucleus-encoded, plastid-targeted GapC genes/enzymes as “GapC1” genes/enzymes according to Liaud et al. (1997) [6]. All GapC1 genes form a robust monophyletic clade in global GAPDH phylogeny including GapC1, GapC, and closely related bacterial homologues [13]. The interpretation of this tree topology was that the GapC1 gene was produced by a single duplication of the gene encoding the cytosolic enzyme followed by changing sub-cellular localization from the cytosol to plastids [9], [10].

Cavalier-Smith (1999, 2002) [14], [15] has proposed that (i) alveolates (including dinoflagellates, ciliates, and apicomplexans), stramenopiles, haptophytes, and cryptophytes — collectively called “chromalveolates” — are monophyletic, (ii) their common ancestor acquired plastids through a single endosymbiosis associated with a red alga, and (iii) multiple lineages in the four groups became secondarily non-photosynthetic (e.g. ciliates). Importantly, it has been widely accepted that the single origin of GapC1 genes is compatible with the monophyly of chromalveolates (e.g. [9], [10]). It is believed that the original GapC1 gene was established in the ancestral chromalveolate cells and was vertically inherited by the extant photosynthetic chromalveolate lineages. However, this simple scenario assuming vertical transfer of the GapC1 genes inevitably confronts serious contradictions. In GapC1 phylogenies, the homologue of the apicomplexan *Toxoplasma* robustly branches with the haptophyte homologues, challenging both host affinity between apicomplexans and dinoflagellates (e.g. [16]), and that between cryptophytes and haptophytes [17]–[19]. In addition, there is a peculiarity regarding GapC1 sequences from stramenopiles. Previously published phylogenies have failed to recover the monophyly of the GapC1 homologues of four stramenopile species, the raphidophycean alga *Heterosigma akashiwo*, the synurophycean alga *Mallomonas rasilis*, and two bacillariophycean algae (diatoms) *Phaeodactylum tricornutum* and *Odontella senensis*, not as would be anticipated from a well established host monophyly of stramenopiles. In order to examine the stramenopile “paraphyly” in GapC1 phylogenies, an improved sampling of stramenopile GapC1 genes is needed. These idiosyncratic aspects in GapC1 phylogeny have implied that the evolution of these unique genes may be more complex than previously thought, but this has not been deeply investigated to date.

In the present study, we determined and identified GapC1 genes from nine stramenopiles and two cryptophytes. By analyzing our GapC1 dataset including 14 homologues from nine stramenopile classes, we have addressed the two issues in the current GapC1 evolution scenario (see above); the incongruity between the gene and host phylogenies, and the stramenopile paraphyly. Based on the results from phylogenetic analyses of the updated GapC1 dataset, we propose a new evolutionary scenario that can explain the idiosyncratic aspects of GapC1 evolution. In contrast to the widely accepted scenario which assumes vertical transfer of GapC1 genes throughout chromalveolate evolution, we speculate that (i) there was a common ancestor of stramenopiles and alveolates as the “innovator” of the original GapC1 gene, and (ii) multiple lateral transfer events have taken place in GapC1 evolution. We also discuss the validity of evaluating the host and plastid evolution in the chromalveolate members by using the GapC1 phylogeny.

## Results and Discussion

### Incongruity between the GapC1 phylogeny and the phylogeny amongst the host lineages bearing GapC1 genes

Previously published GapC1 phylogenies (e.g. [12]) considered only four homologues from three classes of stramenopiles, such as Synurophyceae, Bacillariophyceae, and Raphidophyceae. It may be inadequate to make the four homologues from the three classes represent the diversity of stramenopiles. Aiming for a better coverage of the stramenopile diversity, we experimentally determined new GapC1 genes from four stramenopile species (*Nannochloropsis oculata*, *Haramons dimorpha*, *Olisthodiscus luteus*, and *Vaucheria litorea*) as well as two cryptophytes (*Chroomonas nordstedtii* and *Cryptomonas ovata*) in this study. In addition, six stramenopile GapC1 sequences, which have not been considered in the previously published GapC1 phylogenies, were identified from public sequence databases. Here, we re-examined GapC1 evolution by analyzing a new data set including homologues from nine classes in stramenopiles.

**Figure 1:**
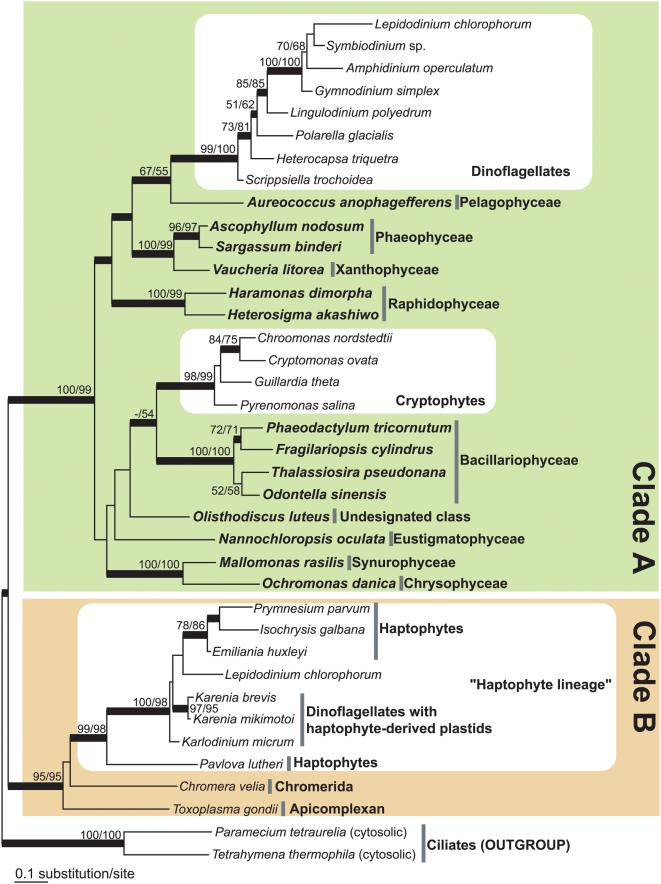
Nuclear-encoded plastid-targeted GAPDH (GapC1) phylogeny. The maximum-likelihood tree was inferred from a GapC1 dataset (38-OTU, 312 amino acid positions) by using RAxML. The tree was rooted by cytosolic GAPDH sequences of two ciliates. The GapC1 tree was divided into two major clades, Clades A and B, highlighted by green and orange shades, respectively. The stramenopile homologues are written with bold letters. ML bootstrap probabilities (RAxML/PhyML) over 50% are shown at the branches. The thick branches represent Bayesian posterior probability over 0.95. Major taxonomic groups are labeled on the right.

In the GapC1 phylogeny shown in Figure 1, all GapC1 homologues considered in this study were separated into two Clades; “A” and “B”: In Clade A, the homologues of photosynthetic stramenopiles, cryptophytes, and “phylogenetically-diverged” dinoflagellates were grouped with 99–100% ML bootstrap values (BP) and 1.00 posterior probabilities (PP). The dinoflagellate homologues and the cryptophyte homologues formed respective monophyletic clades with 98–100% BP and 1.00 PP, and these two clades were separately placed within the radiation of the stramenopile homologues.

The GapC1 homologues from haptophytes, dinoflagellates belonging to the genera *Karenia*, *Karlodinium*, and *Lepidodinium*, the apicomplexan *Toxoplasma*, and the chromerid *Chromera* formed Clade B with 91–95% BP and 1.00 PP. In Clade B, the *Karenia*, *Karlodinium*, and *Lepidodinium* homologues grouped with the homologues of prymnesiphycean haptophytes with 98–100% BP and 1.00 PP. Since the plastids present in *Karenia* and *Karlodinium* are the remnants of an endosymbiotic haptophyte, GapC1 genes from the two dinoflagellate genera are most likely from an endosymbiont (haptophyte) transferred to the host (dinoflagellate) nuclear genome. It has been proposed that *Lepidodinium* with green alga-derived plastids acquired GapC1 gene from a haptophyte in a non-endosymbiotic context [20]. Consequently, the GapC1 homologues from *Karenia*, *Karlodinium*, and *Lepidodinium* can be considered as haptophyte homologues.

The overall GapC1 tree topology shown in Figure 1 agreed with those recovered in previously published studies (e.g. [9]–[12], [20], [21]). However, it has been pointed out that the GapC1 phylogeny is significantly incongruent with the organismal (host) relationships among apicomplexans plus the chromerid *Chromera* (henceforth designated as apicomplexans^+^), dinoflagellates, haptophytes, and cryptophytes widely accepted to date (e.g. [20]). Apicomplexans and dinoflagellates are two out of the three major sub-groups of a large protist assemblage, Alveolata [22]. In “phylogenomic” analyses, the sister relationship between cryptophytes and haptophytes has been consistently recovered [17]–[19]. Nevertheless, the GapC1 phylogeny here recovered neither the host affinity between apicomplexans^+^ and dinoflagellates nor that between cryptophytes and haptophytes (Figure 1). The dinoflagellate homologues were nested in Clade A, while the homologues from apicomplexans^+^ formed Clade B with the haptophyte homologues. Likewise, the cryptophyte and haptophyte homologues were separately included in Clades A and B, respectively. The approximately unbiased (AU) test successfully complemented the ML phylogenetic analysis shown in Figure 1. Alternative tree topologies bearing the monophyly of dinoflagellate and apicomplexan^+^ homologues and the monophyly of the cryptophyte and haptophyte homologues were rejected at the 1% level (*P* = 2×10^−6^ and 0.003, respectively; the details of the alternative trees are shown in Figure S1).

### A new hypothesis for GapC1 gene evolution

The results from our analyses (see above) strongly suggest that GapC1 evolution cannot be explained by any scenarios only invoking vertical transfer of GapC1 genes from the common ancestor of cryptophytes, haptophytes, stramenopiles, dinoflagellates, and apicomplexans^+^. There is extensive literature on lateral transfer of cytosolic GAPDH genes [11], [13], [23], [24], and, intriguingly, GapC1 evolution appears not to be immune from lateral gene transfer (LGT) [20]. Combining the idiosyncratic aspects in the GapC1 phylogeny with “lateral mobility” of GAPDH genes in general, we propose a new hypothesis for GapC1 evolution.

**Figure 2:**
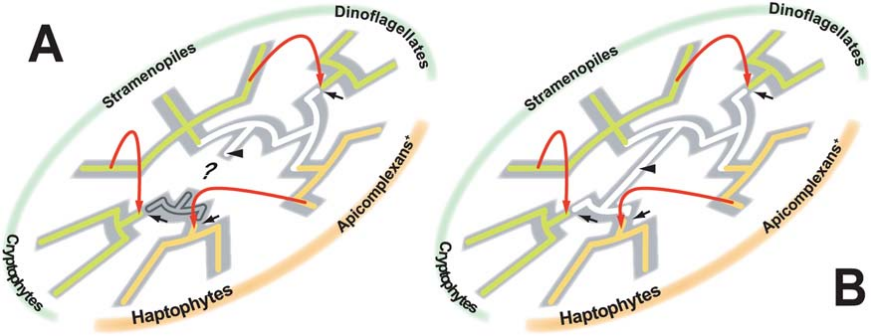
New proposed scheme for GapC1 evolution. A. The original GapC1 gene was established in a common ancestor of stramenopiles and alveolates [including dinoflagellates and aplicomplexans plus *Chromera* (designated as apicomplexans^+^); ciliates are excluded in this figure] shown by an arrowhead. Photosynthetic stramenopiles and apicomplexans^+^ possessed the vertically transferred GapC1 genes. The ancestral dinoflagellates replaced the “vertical” GapC1 gene by a laterally acquired homologue from an unknown stramenopile species. We also assume two lateral GapC1 gene transfer events — one between an unknown stramenopile species and the ancestral cryptophyte cells, and the other between an unknown member of apicomplexan^+^ and the ancestral haptophyte cells. The three LGT events were highlighted by red arrows. Putative replacements of plastid-targeted GAPDH took place after the lateral gene transfers (black arrows). The original type of plastid-targeted GAPDH enzymes in a common ancestor of cryptophytes and haptophytes remains uncertain. The homologues belonging to Clade A in the GapC1 phylogeny (Figure 1) are shown in green, while those belonging to Clade B are shown in orange. The host (or organismal) phylogeny is shown grey shading. In this figure, the host monophyly of haptophytes, cryptophytes, stramenopiles, and alveolates are not assumed. B. The same scheme as shown in A but assuming a host monophyly of haptophytes, cryptophytes, stramenopiles, and alveolates. The original GapC1 gene was established in a common ancestor of the four groups (arrowhead). Under this assumption, a common ancestor of cryptophytes and haptophytes originally utilized the “vertical” GapC1 genes.

If the dinoflagellate and cryptophyte homologues are excluded, Clade A in Figure 1 agrees well with the general view of the stramenopile (host) phylogeny. In Figure 1, monophylies of three classes, Bacillariophyceae (diatoms), Raphidophyceae, and Phaeophyceae, were robustly recovered. In addition, the intimate affinity between Chrysophyceae and Synurophyceae and that between Phaeophyceae and Xanthophyceae were successfully reconstructed as anticipated from phylogenies based on other molecular markers (e.g. [25]). The tree topology of Clade A leads us to a scenario assuming that (i) the homologues of this clade are essentially from stramenopiles, and (ii) the ancestral cells of extant dinoflagellate species and of the extant cryptophytes separately acquired GapC1 genes from two distinctive stramenopile species (schematically shown in Figure 2). The dinoflagellate and cryptophyte clades branched with the *Aureococcus* homologue and the diatom homologue, respectively, although the support for these relationships was inconclusive in ML bootstrap analyses (Figure 1).

Next, we hypothesize the evolutionary process of Clade B in Figure 1 composed of the *Toxoplasma*, *Chromera*, and haptophyte homologues (Figure 1). This unexpected grouping has been suspected to have been produced through LGT (e.g. [10]). In respect of the close (host) relationship between *Toxoplasma* and *Chromera* [26], these homologues should have been robustly grouped, excluding the haptophyte homologues. In reality, in Clade B, the haptophyte clade as a whole was nested within the homologues from *Toxoplasma* and *Chromera* (Figure 1). Thus, the tree topology of Clade B can be reconciled by assuming GapC1 transfer from an unknown member of apicomplexans^+^ to the ancestral haptophyte cells (schematically shown in Figure 2). In this scenario, GapC1 homologues of the extant haptophytes are fundamentally from apicomplexans^+^.

The host sisterhood between stramenopiles and alveolates (including apicomplexans^+^) and the life style of their ancestral cells may hold the key to exploring deeper GapC1 evolution. Firstly, a robust host monophyly of stramenopiles and alveolates has been constantly recovered [16]–[19], [27], [28]. Secondly, many of non-photosynthetic members of stramenopiles and alveolates — oomycetes, *Perkinsus*, *Oxyrrhis*, apicomplexans, and ciliates — still retain relic plastids and/or plastid-derived genes in their nuclear genomes [29]–[36]. These findings suggest that stramenopiles and alveolates evolved from a single, photosynthetic ancestor, and secondary loss of photosynthetic ability (or plastid as a whole) took place in multiple, independent lineages in the two groups. If the origins of the Clade A and Clade B homologues are from stramenopiles and apicomplexans^+^, respectively, the first GapC1 genes may have been established in a common ancestor of stramenopiles and alveolates (arrowhead in Figure 2A). In the subsequent evolution of stramenopiles/alveolates, GapC1 genes may have been lost in secondarily non-photosynthetic lineages.

### Implication for the host and plastid relationships amongst the “chromalveolate” lineages

Photosynthetic stramenopiles, photosynthetic alveolates (including *Toxoplasma*), haptophytes, and cryptophytes utilize GapC1 enzymes for their red alga-derived plastids. This unique molecular “synapomorphy” in the four photosynthetic eukaryotic lineages has prompted a scenario assuming that GapC1 genes were vertically inherited from a common ancestor of these chromalveolate lineages. While this scenario has won popularity, significant incongruity between the GapC1 and host phylogenies has been noticed [20]. Rather, our new hypothesis, in which the incongruity is resolved by invoking LGT, is more favorable than the “standard” hypothesis assuming vertical GapC1 gene transfer in the chromalveolate host evolution. Noteworthy, our hypothesis invoking LGT lends no support to either monophyly or paraphyly of the chromalveolate host lineages, due to no information regarding the original plastid-targeted GAPDH enzymes for cryptophyte and haptophyte plastids. For instance, the putative GapC1 evolution proposed here and the monophyly of chromalveolate host lineages can fit with each other by assuming that cryptophytes and haptophytes originally utilized the GapC1 genes vertically inherited from the ancestral chromalveolates before the putative LGT events (Figure 2B). We consider such “GapC1-to-GapC1” replacements are not unlikely, since a similar event has been already introduced to explain the origin of GapC1 genes in extant dinoflagellates (Figure 2). The uncertainties in the hypothesis for GapC1 evolution discussed above need to be thoroughly re-examined when deeper insights regarding the host and plastid evolution in the chromalveolate lineages are available in the future. At any rate, we recommend splitting the GapC1 evolution and the host evolution of GapC1-containing lineages.

Plastid-encoded gene phylogenies generally support the monophyly of plastids in chromalveolate cells (chromalveolate plastids) (e.g. [37]). On the other hand, the host monophyly of the chromalveolate members has not been validated by any nucleus-encoded gene phylogenies (e.g. [17]–[19]). To reconcile the discrepancy between the chromalveolate host and plastid phylogenies, theories regarding (i) the paraphyly of the chromalveolate host lineages, and (ii) the spread of plastids amongst the chromalveolate lineages via tertiary endosymbioses, have been recently proposed [38]–[40]. However, the GapC1 phylogeny is fundamentally neutral in regard to the theories described above. There is no strong reason to believe that during plastid replacement via tertiary endosymbiosis nucleus-encoded genes for the pre-existing plastids were always replaced by orthologous genes brought by an endosymbiont cell. In fact, the dinoflagellate *Karenia brevis* bearing haptophyte tertiary plastids possesses plastid-targeted genes with phylogenetically diverged origins [41]. A similar phylogenetically chimeric proteome is known from the chlorarachniophyte alga *Bigelowiella natans* [42]. More specifically, the dinoflagellate *Lepidodinium*, which most likely acquired its current plastids from an endosymbiotic green alga, utilizes plastid-targeted GAPDH gene of haptophyte origin [20]. Considering multiple origins of plastid-targeted genes in the nuclear genomes in photosynthetic eukaryotes, we should be aware of the potential “gap” between the evolution of GapC1 genes and that of chromalveolate plastids.

## Materials and Methods

### Algal strains

Two stramenopile species (*Haramonas dimorpha* NIES716 and *Olisthodiscus luteus* NIES15) and two cryptophytes (*Chroomonas nordstedtii* NIES706 and *Cryptomonas ovata* NIES275) were purchased from the Microbial Culture Collection at the National Institute for Environmental Studies (NIES, 16-2 Onogawwa, Tsukuba, Ibaraki 305-8506, Japan). Other stramenopile species, *Vaucheria litorea* CCMP2940 and *Nannochloropsis oculata* CCMP525, were purchased from the Provasoli-Guillard National Center for Culture of Marine Phytoplankton (CCMP: 180 McKown Point Road, West Boothbay Harbor, Maine 04575, USA). These algal cells were grown according to the instructions from CCMP and NIES.

### New plastid-targeted GAPDH sequences

Genomic DNA samples from *Haramonas* and *Olisthodiscus* were prepared by using a SepaGene kit (Sanko Junyaku Co. Ltd., Tokyo, Japan). Total RNA samples from other strains were prepared by using the Absolutely RNA RT-PCR Miniprep Kit (Stratagene, La Jolla, CA, USA) after homogenizing the cell pellets with glass beads in lysis buffer in this kit. Synthesis of cDNA from total RNA was performed using SuperScript III RNase H^−^ reverse transcriptase (Invitrogen, Carlsbad, CA, USA) according to the manufacturer's instructions. PCR amplification using genomic DNA or cDNA as a template was conducted using HotStarTaq DNA polymerase (QIAGEN, Tokyo, Japan). GapC1 genes were amplified using one set of primers (forward: 5′-CCAAGGTCGGNATHAAYGGNTTYGG-3′ and reverse: 5′-CGAGTAGCCCCAYTCRTTRTCRTACCA-3′) [9]. Thermal cycling was comprised of 35 cycles of 0.5–1 min at 94°C, 1 min at 45–50°C, and 2 min at 72°C. The PCR-amplified DNA fragments were cloned into the pCR2.1 vector of the TOPO TA Cloning Kit (Invitrogen). The DNA sequence of each amplified fragment was confirmed with multiple clones. The cytosolic GapC genes were identified from the three species (*Haramons*, *Olisthodiscus*, and *Vaucheria*) (data not shown). The gene sequences determined in the present study have been deposited in the DNA Data Bank of Japan (DDBJ) under accession numbers AB459521–AB459529.

We also identified GapC1 sequences in ongoing-genome and expressed sequence tag (EST) data of four stramenopile species. The GapC1 genes were retrieved from the genome sequence data of the pelagophycean alga *Aureococcus anophagefferens* and the diatom *Thalassiosira pseudonana* (DOE Joint Genome Institue; www.jgi.doe.org). We also identified GapC1 transcripts in the EST data of the phaeophycean alga *Sargassum binderi* and the diatom *Flagilariopsis cylindrus*. The transcripts were assembled into contigs, and the corresponding amino acid sequences were then deduced from the contig sequences.

### Phylogenetic analyses

We manually aligned GapC1 amino acid (aa) sequences from 14 stramenopile species, 11 dinoflagellates, four cryptophytes, four haptophytes, the apicomplexan *Toxoplasma*, and the chromelid *Chromera*, and two cytosolic GAPDH sequences from ciliates (*Parameciuim tetraurelia* and *Tetrahymena thermophila*) as the outgroup. Unambiguously aligned 312 aa positions were retained in the final alignment. This GapC1 dataset was firstly subjected to ProtTest [43] to find the best fit model for ML phylogenetic analyses described below. The “WAG+I+Γ+F” model, in which among-site rate variation was approximated by a discrete gamma distribution plus the proportion of invariant positions, and aa frequencies were estimated from the data, was selected for the GapC1 analyses according to Akaike Information Criterion.

The GapC1 data set was subjected to ML phylogenetic analyses by using RAxML 7.0.4 [44] under the WAG+I+Γ+F model [45]. In the RAxML analyses, the tree search was started from 10 distinct parsimony starting trees. Bootstrap analyses (100 replicates) were conducted as described above except the tree search initiated from a single parsimony tree per replicate. We repeated the ML analysis using PhyML [46] under the WAG+I+Γ+F model. The tree search in the PhyML analysis was started from a BIONJ tree.

The GapC1 data set was analyzed by the Bayesian method by using MrBayes 3.0 [47] under the WAG+I+Γ model, which was the best model selected by ProtTest according to Bayesian Information Criterion. One cold and three heated Markov chain Monte Carlo (MCMC) chains with default-chain temperatures were run for 10^6^ generations, sampling log-likelihoods (InLs), and trees at 100-generation intervals (10^4^ InLs and trees were saved during MCMC). The first 10^5^ generations were discarded as “burn-in”, and Bayesian posterior probabilities and branch-lengths were estimated from the remaining 9×10^5^ generations.

### Approximately unbiased (AU) test

We heuristically searched for (i) the optimal tree with the monophyly of the dinoflagellate and apicomplexan^+^ homologues, (ii) that with the monophyly of the cryptophyte and haptophyte homologues, and (iii) that with both dinoflagellates–apicomplexans^+^ and cryptophytes–haptophytes monophylies, by using RAxML. The details of RAxML tree searches were same as described above. The three alternative trees and the ML tree were subjected to AU test. For each test trees, site-wise log likelihoods (site-lnLs) were calculated by Tree-Puzzle v.7.2 [48]. The resultant site-lnLs data were then input to CONSEL [49].

## Supporting Information

Figure S1 Alternative trees subjected to the AU test. The optimal trees bearing the monophyly of cryptophyte and haptophyte homologues (left) and that bearing the monophyly of the dinoflagellate and apicomplexan+ GapC1 homologues (right) were compared to the ML tree shown in Figure 1 by using the AU test. Branch lengths are ignored in these figures. On the left, the clade of the haptphyte homologues (“H”) and cryptophyte homologues (“C”) is shaded. On the right, the clade of the dinoflagellate homologues (“D”) and apicomplexan+ homologues (“A”) is shaded. The stramenopile homologues are indicated as “S”.
(6.53 MB TIF)
