# Supplementary figures and images for: A Hypothesis for the Evolution of Nuclear-Encoded, Plastid-Targeted Glyceraldehyde-3-Phosphate Dehydrogenase Genes in “Chromalveolate” Members
Source: PLoS One. 2009 Mar 9;4(3):e4737. doi: 10.1371/journal.pone.0004737 (PMC2649427; doi:10.1371/journal.pone.0004737)

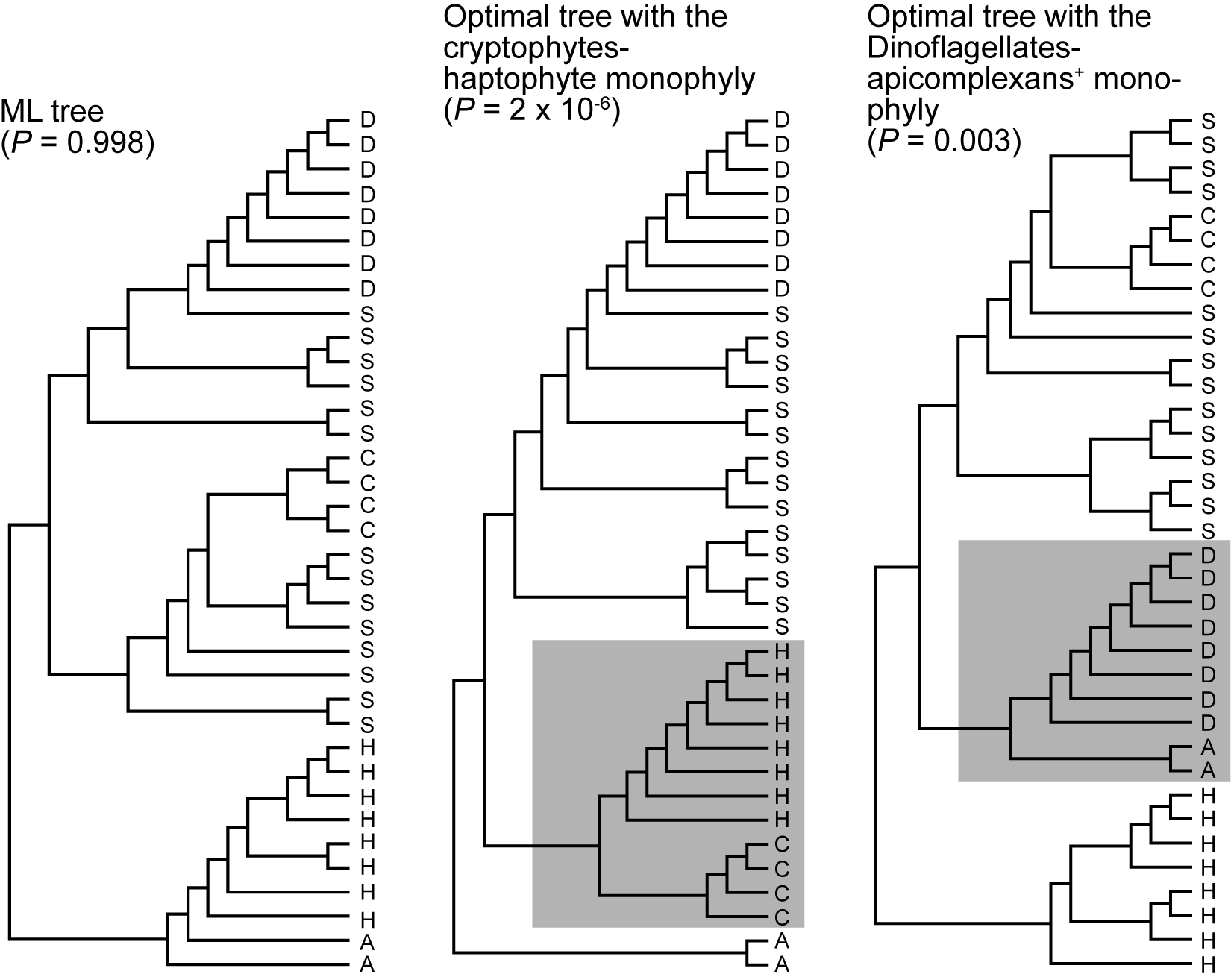

Supplement: Figure S1 — Alternative trees subjected to the AU test. The optimal trees bearing the monophyly of cryptophyte and haptophyte homologues (left) and that bearing the monophyly of the dinoflagellate and apicomplexan+ GapC1 homologues (right) were compared to the ML tree shown in Figure 1 by using the AU test. Branch lengths are ignored in these figures. On the left, the clade of the haptphyte homologues (“H”) and cryptophyte homologues (“C”) is shaded. On the right, the clade of the dinoflagellate homologues (“D”) and apicomplexan+ homologues (“A”) is shaded. The stramenopile homologues are indicated as “S”. (6.53 MB TIF) [file pone.0004737.s001.tif]
